# Knowledge and practice on prevention of mosquito-borne diseases in livestock-keeping and non-livestock-keeping communities in Hanoi city, Vietnam: A mixed-method study

**DOI:** 10.1371/journal.pone.0246032

**Published:** 2021-02-04

**Authors:** Thang Nguyen-Tien, Long Thanh Pham, Duoc Trong Vu, Son Hai Tran, Lieu Thi Vu, Vuong Nghia Bui, Anh Ngoc Bui, Trung Duc Hoang, Thanh Thi Vu, Hung Nguyen-Viet, Ulf Magnusson, Åke Lundkvist, Johanna Lindahl

**Affiliations:** 1 Department of Medical Biochemistry and Microbiology, Zoonosis Science Center, Uppsala University, Uppsala, Sweden; 2 International Livestock Research Institute, Hanoi, Vietnam; 3 National Institute of Hygiene and Epidemiology, Hanoi, Vietnam; 4 National Institute of Veterinary Research, Hanoi, Vietnam; 5 Hanoi University of Public Health, Hanoi, Vietnam; 6 Department of Clinical Sciences, Swedish University of Agricultural Sciences, Uppsala, Sweden; University of Liverpool, KENYA

## Abstract

Mosquito-borne diseases (MBDs) are causing high morbidity and mortality for humans. Urban livestock keeping is still common in cities around the world. The animals may serve as reservoirs for zoonotic MBDs, which increase the risks for humans. Here we assess the knowledge and practices related to MBDs in households with livestock and without livestock and explore the perceptions of the health care sector about MBDs and livestock keeping in Hanoi city of Vietnam in a cross-sectional study. A quantitative survey was conducted including 513 households with and without livestock-keeping in six districts and complemented with qualitative surveys with four health staff from Hanoi Center of Disease Control and three district health centers. The quantitative survey indicated that the participants possessed basic knowledge on MBDs with an average score of 18.3 out of 35, of which non-livestock-keeping households had a better knowledge than households keeping livestock (p<0.05). Both household categories had low score, 3.5 out of 11, regarding preventive practices against MBDs. The negative binomial model showed that occupation and location of living were factors associated to the knowledge on MBDs. Farmers were likely to have better preventive practices as compared to office workers (p<0.05). Those who had better knowledge also had more adequate preventive practices against MBDs (p<0.001). The qualitative survey revealed that livestock keeping was determined as increasing risks of MBDs due to the increase of mosquito population. It is recommended that community campaigns to raise the awareness and change behavior on MBDs should be organized based on collaboration between the health sector and the veterinary sector for households with and without livestock living in central urban and peri-urban areas. Further studies are needed to confirm the association between urban livestock keeping and potential increasing risks of MBDs such as dengue and Japanese encephalitis.

## Introduction

Mosquito-borne diseases (MBDs) are increasing problems in tropical cities where fast urbanization, with migration and population growth, is occurring [1]. These diseases are widely spread across the world, however, concentrated mainly to tropical countries with hot and humid climates. MBDs previously thought to be under control, such as dengue fever (DF) and Japanese encephalitis (JE) are now re-emerging and the introduction of viruses such as Zika virus in new areas poses an increasing global public health threat [2, 3]. No vaccines are yet available for the majority of MBDs. Therefore, prevention relies mainly on vector control through environmental management and change of behaviour [4]. However, the effectiveness of such methods may be limited due to lack of understanding, funding, and/or engagement from the authorities and communities [5]. In addition, earlier studies have indicated that low levels of knowledge and poor practice of preventive measures against MBDs in communities may increase the morbidity [6].

Keeping of livestock in urban places is common as a way to provide urban inhabitants with perishable high value food products and to create opportunities for livelihoods [7]. However, the livestock can attract vectors through odors and become potential blood meal sources for some mosquitoes. Moreover, livestock keeping may also cause more water sources to be around, enabling mosquito breeding. It is thereby hypothesized that livestock-keeping may increase vector populations, leading to increasing risk of mosquito-borne infections of humans. There is conflicting evidence for this hypothesis. Studies in southern Vietnam found that livestock-keeping was associated with increasing numbers of the mosquito-vector of JEV [8, 9]. Another study in Vietnam indicated that having an animal shelter correlated to a higher incidence of dengue fever [6]. Seyoum et al. also reported that the presence of cattle-keeping in homesteads tends to increase the man-biting rate of *Anopheline* mosquitoes in Ethiopia [10]. However, a recent study of Jakobsen et al. conducted in Hanoi city, Vietnam, could not find evidence that households with livestock were at higher risk of getting dengue fever as compared to non-livestock households [11].

With a population of around 8 million people, Hanoi is Vietnam’s second largest city and experiences urbanization at a considerable rate. It has a warm and humid sub-tropical climate with four seasons. In addition to climate changes, massive population growth along with unplanned urbanization is considered an important contributor to increase the density of vectors for MBDs, causing more frequent outbreaks of MBDs, particularly dengue, in cities [12, 13]. According to the General Statistics Office (GSO), the livestock in Hanoi is diverse and numerous, with around 157500 cattle, 1635900 pigs and 25620000 poultry reported in 2018 [14]. Since livestock keeping in the urban areas can be a risk factor of getting MBDs through growing mosquito populations, this study aimed to assess the level of knowledge and practice related to prevention and control of MBDs in the livestock-keeping and non-livestock-keeping community and explore the perception from healthcare sector about MBDs and livestock keeping in Hanoi city, Vietnam.

## Materials and methods

### Study design

A cross-sectional study using sequential explanatory mixed methods (the data were collected in two consecutive phases, of which quantitative data were collected and analysed first. Qualitative data were collected in the second phase of the study and were related to the outcomes from the quantitative data) was performed. Quantitative data were collected in the first phase from September to October 2018. Qualitative data were collected to complement to the quantitative results in December 2018 in the second phase.

### Study sites

**Fig 1:**
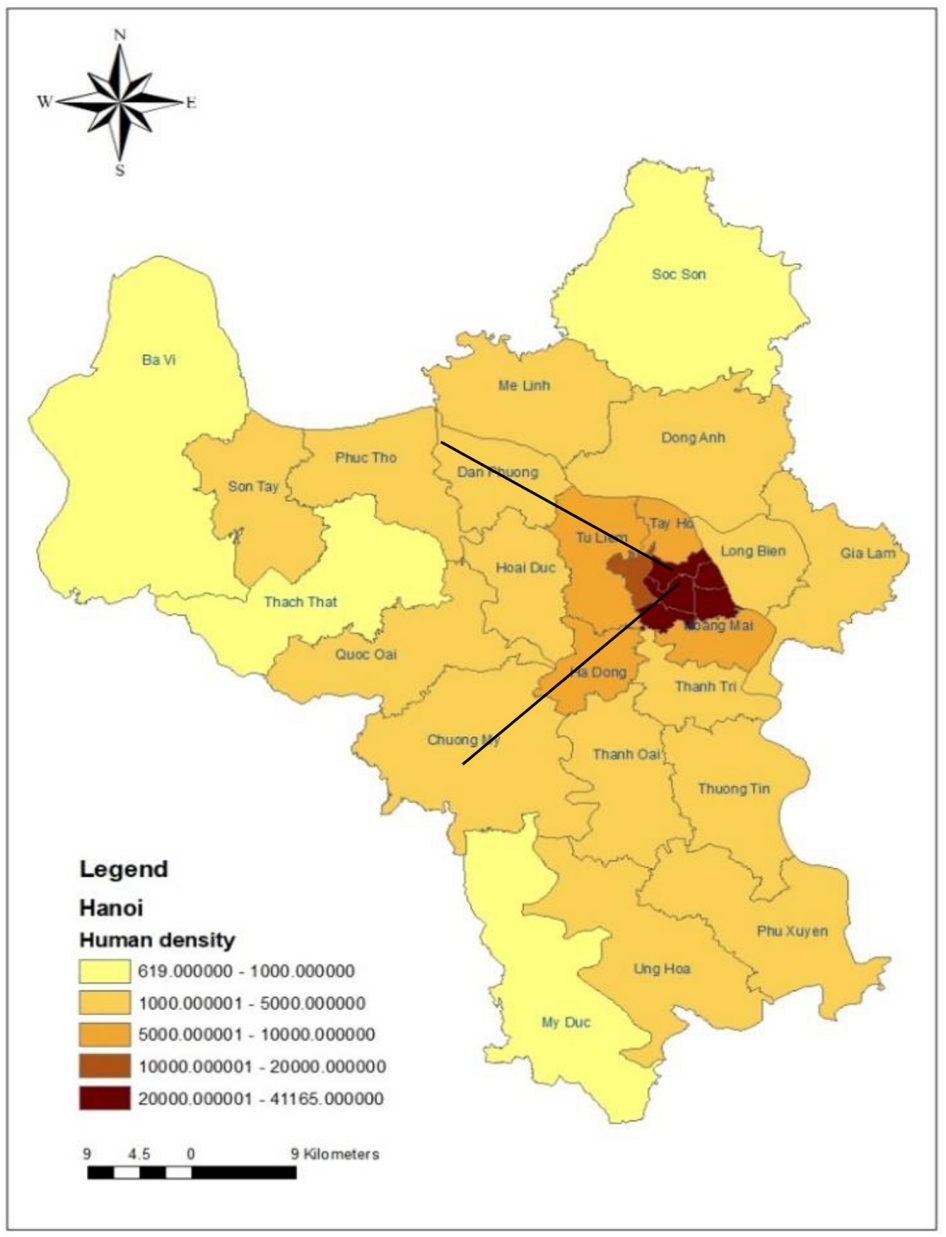
Map of Hanoi with transects through the study districts.

Maps of Hanoi city were created using ArcGIS version 10.3 ArcMap (ESRI, Redlands, CA), and two transects were added (Fig 1), from the city centre to the peri-urban parts of the province. Six districts were selected in these transects; two urban central districts which comprises the inner districts of old Hanoi where no livestock is kept, namely *Ba Dinh* and *Cau Giay;* two peripheral districts that are newly expanded districts of new Hanoi where some livestock are kept (below 300000 poultry animals and 100000 large ruminants and pigs, namely *Ha Dong* and *Bac Tu Liem*; and two peri-urban districts comprises suburban districts where large livestock populations are kept (above 300000 poultry animals and 100000 large ruminants and pigs), namely *Chuong My* and *Dan Phuong*.

### Quantitative survey

#### Population, sampling and sample size

**Fig 2:**
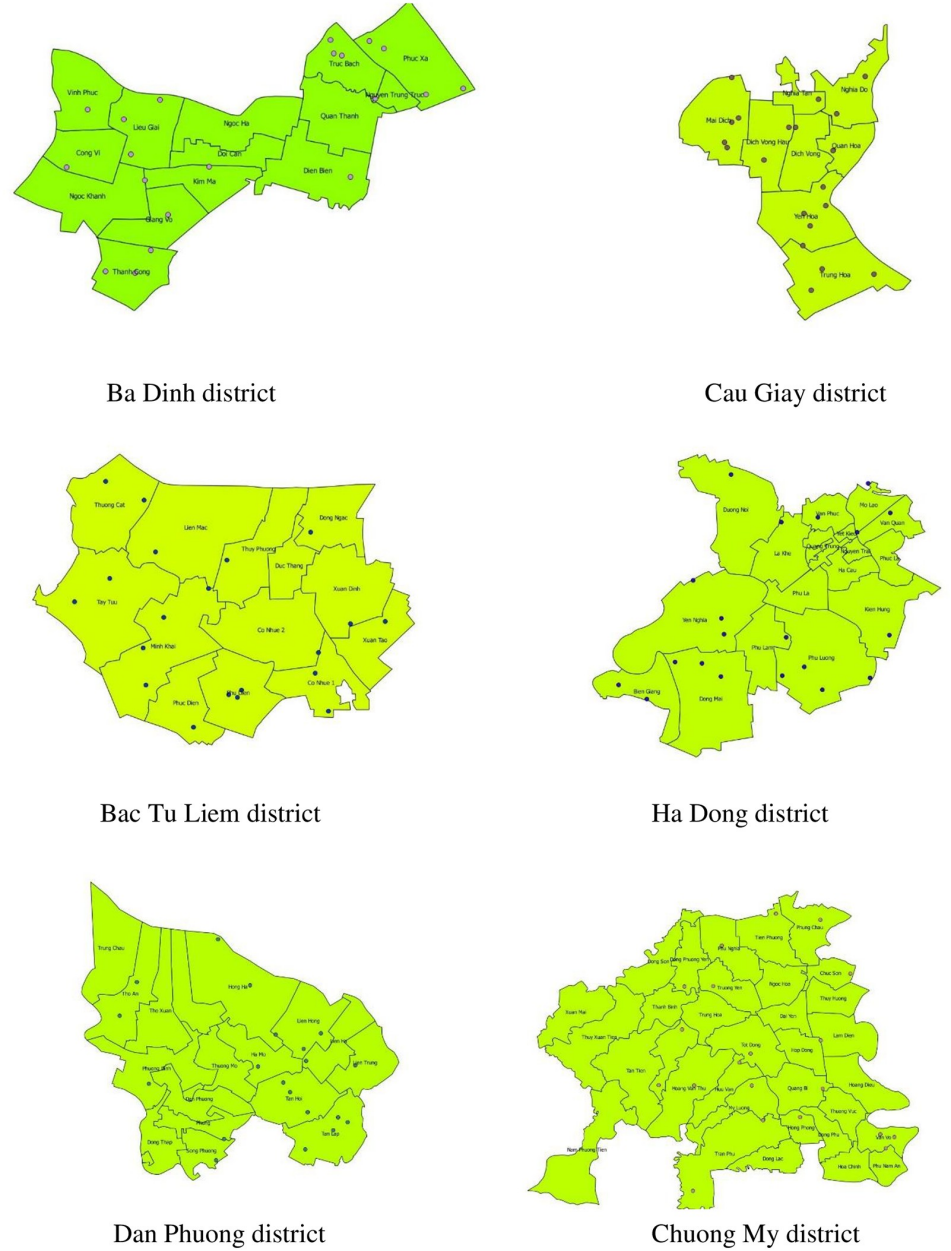
GPS points in 6 districts.

In the selected six districts, the households were selected using randomly generated GPS-points in ArcGIS version 10.3 ArcMap (ESRI, Redlands, CA) (Fig 2). From each GPS-point, three livestock households were identified and approached in three different directions within a radius of 2 km, and three other non-livestock households nearby. Thus, six households were chosen for each randomly selected point. If the first household was not willing to participate in the study, the interviewer continued until a consenting household was found.

A calculation of sample size was performed by assuming a power of 0.8, alpha 0.05, and a desire to detect a difference of 20% between households with livestock keeping compared to households without livestock keeping (STATA power two proportion 0.5 & 0.7); 93 households in each category were needed (with and without livestock in each strata of districts). In order to account for clustering and multiple comparisons, the sample size was increased by 10%, giving us a total sample size in each category of 102. Because two central districts did not keep livestock, therefore, we only included households without livestock. In total, **513 households** were recruited in the quantitative survey.

A household with livestock was defined as a household having at least one larger livestock species (pig, cattle, goat or larger); or at least 5 smaller food-producing animals (chicken, rabbits, dogs, duck, goose), meaning at least 0.05 tropical livestock units [15].

The household owner or one adult (above 18) person living permanently in the household was interviewed using a questionnaire.

#### Data collection

A questionnaire from a previous study was adapted and improved [11]. The questionnaire (Supporting information 3) included four sections with a number of multiple-choice and single-choice questions: (i) Demographic characteristics of the respondent (ten questions), (ii) livestock information (eight questions); (iii) awareness about MBDs (seven questions on knowledge with 39 items and one question on practice with 12 items), (iv) sources of information that participants have heard about the MBDs (two questions). Since households with and without livestock may have different socioeconomic circumstances, we included collection of income and education. The district veterinary staff were trained to use the paper-based questionnaires to interview the householders in Vietnamese. The face-to-face interviews took place in the households and lasted around 20 minutes.

#### Data analysis

For the quantitative data analyses, Epidata (EpiData Association, Odense Denmark) was used for entry and data were transfer to STATA 15.0 (STATACorp Ltd, College station, Texas) for management and analysis.

Cronbach Alpha was used to test the internal consistency of the awareness about MBDs. The results were 0.89 for 39 knowledge items and 0.72 for 12 practice items, indicating high consistency. In order to assess the knowledge of participants, their responses were marked by zero or one points in correspondence to the accurate and inaccurate answers to each question. The total score of all questions about names of various MBDs, breeding sites of mosquitoes, risk factors of MBDs, symptoms of MBDs, and preventive measures of MBDs were calculated. The knowledge score ranged from 0–35 points. The practice score was based on the sum of all methods that the participants have used to prevent and control MBDs. The practice score ranged from 0–11 points. Participants that acquired higher scores were supposed to have better levels of knowledge and practice.

Chi-square, Fisher exact and Mann-Whitney tests were used to compare the differences between households with livestock and without livestock. Since the knowledge and practice scores were not distributed normally, Spearman’s rho was used to describe the relationship between knowledge score and practice score. The Mann-Whitney test, the Kruskal Wallis test and negative binomial regression were used to identify factors associated with knowledge and practice scores. Factors with a p-value less than or equal to 0.1 in the tests were included in the multivariable negative binomial model. A p-value less than 0.05 was considered statistically significant.

### Qualitative survey

#### Sample size

Five households with livestock (two households in *Chuong My district*, one households in *Dan Phuong district*, one household in *Bac Tu Liem district* and one household in *Ha Dong district*) and five households without livestock (two households in *Ba Dinh district*, one household in *Cau Giay district*, one household in *Bac Tu Liem district*, one household in *Ha Dong district*) in six districts were conveniently chosen from those included in the survey, based on their availability for person-to-person in-depth interviews.

Additionally, we had in-depth interviews with one representative from the Department of Vector-borne Disease of Hanoi Preventive Medicine Center (now called Hanoi Centre of Disease Control) and three representatives from the health sector who worked in communicable diseases and/or vector surveillance in three categorized district health centers including *Ba Dinh*, *Ha Dong* and *Chuong My*.

#### Instruments

Two in-depth interview guidelines (Supporting information 4) were constructed to gain deeper understanding on the MBDs’ perception of households and health staffs in the livestock areas and non-livestock areas.

#### Data collection

The first two authors (TNT & LPT) were responsible for conducting qualitative interviews. All in-depth interviews were in Vietnamese and lasted approximately 15 to 25 minutes. All discussions were conducted at participants’ household or office and tape-recorded with their permission. The recorded files were re-listened to check the quality and sent for transcription within 48 hours.

#### Data analysis

All discussions were recorded and transcribed verbatim. Data was analyzed by coding and categorizing. The first author performed content analysis by organizing all interview texts into key themes that was double-checked by the last author. Only the quotations shown as illustrations for the findings were translated to English.

### Ethical statement

Ethical approval for this research has been obtained from the Ethical Committee of Hanoi University of Public Health (No 406/2018/YTCC-HD3). All human subjects were informed about the study purposes and asked to sign their name in a written consent form if they agreed to participate voluntarily.

## Results

Socio-demographic characteristics of the participating households are shown in Table 1.

**Table 1:** Socio-demographic characteristics of the participating households with and without livestock in Hanoi, Vietnam.

	*Households with livestock (n/%)*	*Households without livestock (n/%)*	*All (n/%)*
***Respondent gender***			
Male	178 (76.4%)	179 (63.9%)	357 (69.6%)
Female	55 (23.6%)	101 (36.1%)	156 (30.4%)
***Respondent age***			
18–30	2 (1%)	14 (5.8%)	16 (3.6%)
31–45	67 (33.3%)	58 (24.1%)	125 (28.3%)
46–60	98 (48.7%)	106 (43.9%)	204 (46.1%)
>60	34 (16.9%)	63 (26.1%)	97 (21.9%)
***Respondent education level***			
Primary school	18 (8%)	12 (4.6%)	30 (6.2%)
Secondary school	116 (51.5%)	66 (25.5%)	182 (37.6%)
High school	73 (32.4%)	116 (44.8%)	189 (39.1%)
College/University	18 (8%)	65 (25.1%)	83 (17.1%)
***Respondent marriage status***			
Single	2 (0.9%)	15 (5.5%)	17 (3.4%)
Married	226 (97.8%)	250 (92.6%)	476 (95%)
Separated/Divorced	2 (0.9%)	2 (0.7%)	4 (0.8%)
Widowed	1 (0.4%)	3 (1.1%)	4 (0.8%)
***Respondent main occupation***			
Office worker	11 (4.9%)	43 (16%)	54 (11%)
Farmer	173 (77.6%)	84 (31.3%)	257 (52.3%)
Unemployed	13 (5.8%)	20 (7.5%)	33 (6.7%)
Retired	9 (4%)	66 (24.6%)	75 (15.3%)
Other	17 (7.6%)	55 (20.5%)	72 (14.6%)
***Sources of water used in the family***			
Tap water	127 (56.7%)	207 (79.3%)	334 (68.9%)
Rain	50 (22.3%)	34 (13%)	84 (17.3%)
Rivers/lakes nearby	2 (0.9%)	0	2 (0.4%)
Well	156 (69.6%)	67 (25.7%)	223 (45.9%)
***Average income (million VND)***			
**Mean score ± SD**	9.3 ± 0.49	11.2 ± 0.57	10.3 ± 0.38

Males accounted for the majority of the participants (69.6%). Almost half of the participants (46.1%) were 46–60 years old, followed by the age-group of 31–45 years (28.3%). Regarding educational levels, 39.1% of the participants had completed high school, while 37.6% of the participants had completed secondary school. There were more participants completing high school and college/university from households without livestock than from households with livestock. Over half of all respondents had farming (52.3%) as their principal occupation. There were more respondents that were farmers in households with livestock than in households without livestock, whereas there were more office workers and retired participants in households without livestock. The majority of respondents were married (95%). The households without livestock (79.3%) were much more likely to use tap water than the households with livestock (56.7%). In contrast, households with livestock utilized were two times more likely to utilize water from well than the households without livestock. Households without livestock had higher average incomes than households with livestock.

### Households’ knowledge

Table 2 presents the participants’ knowledge on mosquitoes and MBDs of households with or without livestock. Most participants (98%) had previously heard about MBDs, with more households without livestock having heard about MBDs as compared to households with livestock (p<0.05). The most common MBD that the respondents had previously heard of was dengue (96.5%), followed by malaria (72.1%). Participants from households without livestock that knew about malaria was much more common than from households with livestock (p<0.01). Fewer participants (14.4%) had heard about JE and only 1.8% had heard about filariasis.

**Table 2:** Knowledge about mosquitoes and mosquito-borne diseases in households with and without livestock in Hanoi, Vietnam.

	*Households with livestock (n/%)*	*Households without livestock (n/%)*	*All (n/%)*	*P value*
***Heard about MBDs***				
Yes	222 (96.5%)	272 (99.3%)	492 (98%)	**P = 0.028**
No	8 (3.5%)	2 (0.7%)	10 (1.9%)	
***Heard about each disease***				
Dengue fever	216 (96.8%)	259 (96.3%)	475 (96.5%)	P = 0.72
Japanese Encephalitis	25 (11.2%)	46 (17.1%)	71 (14.4%)	P = 0.06
Zika infection	86 (38.6%)	99 (36.8%)	185 (37.6%)	P = 0.68
Malaria	148 (66.3%)	207 (76.9%)	355 (72.1%)	**P = 0.009**
Filariasis	5 (2.2%)	4 (1.5%)	9 (1.8%)	P = 0.53
***Breeding sites of mosquitoes***				
Don’t know	3 (1.3%)	1 (0.3%)	4 (0.8%)	P = 0.33
Clean water	42 (18.5%)	50 (18.5%)	92 (18.5%)	P = 0.99
Drain/polluted water	183 (80.6%)	214 (79.2%)	397 (79.9%)	P = 0.7
Stagnant water containers	183 (80.6%)	236 (87.4%)	419 (84.3%)	**P = 0.038**
Car tires	72 (31.7%)	124 (45.9%)	196 (39.4%)	**P = 0.001**
Water tanks	163 (71.8%)	191 (70.7%)	354 (71.2%)	P = 0.79
Vase	83 (36.6%)	134 (49.6%)	217 (43.6%)	**P = 0.003**
Bonsai rockery	81 (35.7%)	132 (48.9%)	213 (42.8%)	**P = 0.003**
***Risk factors for getting MBD***				
Don’t know any	13 (5.7%)	6 (2.2%)	19 (3.8%)	**P = 0.042**
Warm and humid season	173 (76.2%)	227 (84.1%)	400 (80.5%)	**P = 0.028**
High population density	68 (29.9%)	90 (33.3%)	158 (31.8%)	P = 0.421
Stagnant water	163 (71.8%)	190 (70.4%)	353 (71%)	P = 0.725
Livestock keeping	99 (43.6%)	69 (25.5%)	168 (33.8%)	**P<0.001**
***Season that MBDs are highest***				
Rainy season	211 (92.5%)	249 (91.9%)	460 (92.2%)	P = 0.23
Dry season	1 (0.4%)	6 (2.2%)	7 (1.4%)	
Same	16 (7%)	16 (5.9%)	32 (6.4%)	
***Symptoms while getting MBDs***				
Don’t know any	17 (7.4%)	11 (4%)	28 (5.6%)	P = 0.09
High fever	203 (89%)	256 (94.1%)	459 (91.8%)	**P = 0.04**
Muscle pains	73 (32%)	118 (43.4%)	191 (38.2%)	**P = 0.009**
Nausea/vomiting	71 (31.1%)	104 (38.2%)	175 (35%)	P = 0.09
Severe headache	99 (43.4%)	124 (45.6%)	223 (44.6%)	P = 0.62
Rash	48 (21%)	54 (19.8%)	102 (20.4%)	P = 0.74
Hemorrhage	132 (57.9%)	163 (59.9%)	295 (59%)	P = 0.645
***Ways to prevent getting MBDs***				
Don’t know any	4 (1.7%)	0	4 (0.8%)	**P = 0.043**
Screening of doors/windows	33 (14.5%)	77 (28.3%)	110 (22%)	**P = 0.001**
Mosquito repellent creams/liquid	84 (36.8%)	132 (48.5%)	216 (43.2%)	**P = 0.009**
Mosquito nets	221 (96.9%)	262 (96.3%)	483 (96.6%)	P = 0.7
Electric rackets	135 (59.2%)	174 (63.9%)	309 (61.8%)	P = 0.275
Mosquito coils/Incense sticks	91 (39.9%)	114 (41.9%)	205 (41%)	P = 0.65
Long sleeves	84 (36.8%)	113 (41.5%)	197 (39.4%)	P = 0.28
Keep lids on water tanks	95 (41.7%)	127 (46.7%)	222 (44.4%)	P = 0.26
Chemical in water containers	15 (6.6%)	31 (11.4%)	46 (9.2%)	P = 0.063
Anti-mosquito products (e.g. insecticides)	131(57.4%)	145 (53.3%)	276 (55.2%)	P = 0.353
Eliminate breeding sites	83 (36.4%)	74 (27.2%)	157 (31.4%)	**P = 0.027**
Using fish in water containers	108 (47.4%)	137 (50.4%)	245 (49%)	P = 0.5
**Mean score ± SD**	***17*.*8 ± 6*.*9***	***18*.*7 ± 6*.*4***	***18*.*3 ± 6*.*6***	**P = 0.048**

Qualitative findings indicated that all households with or without livestock could mention DF as the most severe MBD in Hanoi. However, their attitude about the seriousness of MBDs in general, and DF in particular, varied.

*“Dengue is the most regular disease*, *I think*, *my family haven’t had it before but I’m afraid of the dengue mosquitoes*. *It is a dangerous disease*, *can lead to death*

- IDI with household without livestock in Ba Dinh district.

*“Dengue is a serious disease; people are scared of it because it transmits very fast*. *Although it doesn’t cause many deaths but who knows”*

- IDI with household with livestock in Dan Phuong district.

*“I didn’t get infection before*, *so I don’t care much about the disease”*

- IDI with household without livestock in Cau Giay district.

*“Around the area I’m living*, *I don’t see any cases*, *so I think it’s not a serious problem”*

- IDI with household with livestock in Chuong My district.

A large number of participants of households with and without livestock recognized stagnant water containers (84.3%) and tanks (71.2%) as sites for mosquitoes laying eggs. More participants of household without livestock than households with livestock could list stagnant water containers, car tires, vases and bonsai rockery as potential breeding sites of mosquitoes (p<0.05). Nearly 80% of the participants responded that mosquitoes can breed in drain/polluted water, while only 18.5% of them knew that mosquito also can lay eggs in clean water. In terms of the risk factors for getting MBDs, more than 96% of the respondents could list at least one factor. More households with livestock could not identify any risk factors of getting MBDs than households without livestock (p<0.05). Warm and humid weather was considered a risk factor of MBDs by 80.5% of respondents. The percentage of households without livestock that knew about warm and humid weather as a risk factor of MBDs was significantly higher as compared to households with livestock (p<0.05). More households with livestock (43.6%) thought that livestock rearing activities could increase the risk of getting MBDs than households without livestock (25.5%). This difference was statistically significant (p<0.001). 92.2% of the respondents identified correctly that the rainy season is the time period with the highest risk of suffering of MBDs.

The findings from in-depth interviews also showed that the participants could mention a variety of risk factors for MBDs that will increase the number of mosquitoes at home such as stagnant water in leaves, containers, weather, urbanization (pending construction sites, abandoned houses, etc), low awareness of citizen and livestock keeping.

*“Some people plants too much trees without pruning or leave the standing water in uncovered containers and tanks*. *It leads to the growth of mosquitoes”*

- IDI with household without livestock in Ba Dinh district.

*“It depends much on the weather*. *A lot of mosquitoes will appear in the humid weather after Tet holidays…Keeping livestock will have more mosquitoes than no livestock keeping*. *Simple speaking*, *it’s different between animal shelter area and space inside the house*. *There are less mosquitoes inside my house”*

- IDI with household with livestock in Dan Phuong district.

*“Many students are not keeping clean hygiene*. *Or no one discard the garbage in the abandoned houses or unfinished constructing houses*. *Mosquitoes came from there*, *I suppose”*

- IDI with household without livestock in Bac Tu Liem district.

*“It is certain that rearing pigs and chickens can increase the amounts of mosquitoes*”

- IDI with household with livestock in Chuong My district.

Regarding the symptoms of MBDs, most respondents knew that high fever is a prominent symptom when getting MBDs (91.8%). However, the number of households without livestock that could mention this symptom was statistically higher than the number of households with livestock (p<0.05). 5.6% of respondents did not know any symptoms of MBDs. Other possible symptoms including muscle pains, nausea/vomiting, severe headache, rashes and hemorrhages were less mentioned in both households with and without livestock, ranging from 20.4% to 59%. The respondents of households without livestock were more likely to mention muscle pains as a symptom of MBDs than the respondents of households with livestock (p<0.01).

In regard to their knowledge on preventive measures of MBDs, all participants of households without livestock knew one or more methods to prevent mosquito bites, whilst 1.7% of the participants of households with livestock could not mention any (p<0.05). The percentage of households without livestock knowing about the preventive measures of screening of doors/windows and mosquito repellent creams/liquid was statistically higher than households with livestock (p<0.01). In contrast, elimination of breeding sites was much more known by households with livestock than households without livestock with p<0.05. The majority of the participants (96.6%) considered mosquito nets as an important way to prevent MBDs. Other protective measures including using electric rackets, mosquito coils /incense sticks, lids on water tanks, anti-mosquito products, fish in the water containers, or wearing long sleeves were also mentioned, ranging from 39.4% to 61.8%. Using chemical in water containers was the least known (9.2%).

In summary, households without livestock had a higher knowledge score than households with livestock (p<0.05). This finding was consistent with the qualitative results when some households with livestock considered themselves lacking knowledge on MBDs, whereas some households without livestock did know how mosquitoes may transmit diseases to people.

*“We don’t know about the mosquito-borne diseases*, *we were only heard about its name and reminded to spray when the outbreaks did occur”*

- IDI with household with livestock in Chuong My district.

*“I know that mosquitoes suck blood from person with dengue fever then bite another person*. *That person will get the disease”*

- IDI with household without livestock in Ba Dinh district.

### Households’ practices on prevention of MBDs

Table 3 reveals that all households conducted at least one personal protection to prevent MBDs at home. The measure which respondents used most was mosquito nets (90.5%). The percentage of households with livestock used nets for preventing mosquito bites were higher than the percentage of households without livestock (p<0.01). Approximately half of respondents had used electric rackets (51.3%) and anti-mosquito products (49.3%) to kill mosquitoes. Some preventive methods were less used by the respondents ranging, including mosquito repellent creams/liquid; mosquito coils; eliminate breeding sites; using fish in water containers and keep lids on water tanks. The least common methods were using chemicals in water containers and screening of windows/doors. Remarkably, the percentage of households with livestock doing some preventive measures against MBDs was significantly higher than households without livestock including using mosquito nets, using mosquito coils/incense sticks, using anti-mosquito products, eliminating breeding sites and using fish in water containers (p<0.05). However, overall, there was no significant difference between the practice score of households with and without livestock. Although, there was an opinion that households with livestock prevent MBDs better than households without livestock in the same living area.

**Table 3:** Participants’ practice on prevention against MBDs in households with and without livestock in Hanoi, Vietnam.

*Personal protection used to prevent mosquito-borne diseases*	*Households with livestock*	*Households without livestock*	All *(n*, *%)*	P value
Don’t use any measures	0	0	0	
Screening of windows/doors	14 (6.2%)	28 (10.4%)	42 (8.5%)	P = 0.09
Mosquito repellent creams/liquid	32 (14.1%)	48 (17.8%)	80 (16.1%)	P = 0.26
Mosquito nets	215 (95.1%)	233 (86.6%)	448 (90.5%)	**P = 0.001**
Electric rackets	117 (51.8%)	137 (50.9%)	254 (51.3%)	P = 0.852
Mosquito coils/incense sticks	53 (23.4%)	30 (11.1%)	83 (16.8%)	**P<0.001**
Long sleeves	62 (27.4%)	79 (29.4%)	141 (28.5%)	P = 0.63
Keep lids on water tanks	76 (33.6%)	80 (29.7%)	156 (31.5%)	P = 0.35
Chemical in water containers	8 (3.5%)	12 (4.4%)	20 (4%)	P = 0.6
Anti-mosquito products/Insecticide	124 (54.9%)	120 (44.6%)	244 (49.3%)	**P = 0.023**
Eliminate breeding sites	72 (31.8%)	58 (21.5%)	130 (26.2%)	**P = 0.01**
Using fish in water containers	77 (34.1%)	66 (24.5%)	143 (28.9%)	**P = 0.02**
**Mean score ± SD**	3.7 ± 2.4	3.3 ± 2.1	3.5 ± 2.3	P = 0.11

*“I suppose that household with livestock like us frequently keep hygiene in the animal shelter*, *I guess we even have less mosquitoes than household without livestock”*

- IDI with household with livestock in Chuong My district.

Qualitative interviews showed that the households used many preventive practices against MBDs such as mosquito net, coil, electric mosquito trap, fish in the bonsai, clean the house and remove the standing water, spraying. etc. The households with livestock mostly mentioned about spraying disinfectants at their animal shelter by the veterinary staffs or themselves to prevent the mosquito growth.

*“My family uses the electric trap made in Thailand; it can kill a lot of mosquitoes*… *I also have to remove the water in flower vases frequently”*

- IDI with household without livestock in Ba Dinh district.

*“I sprayed frequently at home and animal shelter*. *I think that was the most efficient way to get rid of mosquitoes”*

- IDI with household with livestock in Dan Phuong district.

*“I got free spraying from veterinary staffs to spray at my animal shelter*. *I also bought the anti-mosquito product to spray more*, *so the mosquito was less”*

- IDI with household with livestock in Bac Tu Liem district.

Nevertheless, several participants thought that the preventive practices should be implemented by local authority. In addition, many admitted to sometimes not using protection against MBDs.

*“That practice (insecticide spraying) is the work of ward people’s committee*, *the families can’t do that”*

- IDI with household without livestock in Bac Tu Liem district.

*“I slept without mosquito nets many times*, *in the noon time or sometimes in the night because I didn’t see the mosquitoes*”

- IDI with household with livestock in Ha Dong district.

### Correlation between knowledge and practices

Spearman test indicated an overall positive correlation between knowledge and practice (Spearman’s rho = 0.67, p < 0.001). Classified by livestock keeping, there was a strong positive correlation (r = 0.73, p<0.001) between knowledge and practice of non-livestock households and positive correlation (r = 0.58, p<0.001) between knowledge and practice of livestock households.

### Associated factors with knowledge and practice about MBDs

Table 4 shows the factors associated with knowledge and practice, respectively, about MBDs. Compared to households in peri-urban districts, households living in central urban districts were more likely to have better knowledge regarding MBDs (p = 0.016). Being farmers, unemployed and retired people correlated to a poorer knowledge on MBDs as compared to those working as office workers with p<0.05. Interestingly, compared to office workers, the farmers were more likely to have better preventive practices on MBDs (p = 0.044), in spite of poorer knowledge. People who had higher knowledge score also were more likely to have higher score of preventive practices (p<0.001). There was no significant difference of knowledge and practice scores of households with and without livestock keeping.

**Table 4:** Associated factors with knowledge and practice about MBDs.

	Knowledge score			Practice score		
	Coefficient	CI 95%	P value	Coefficient	CI 95%	P value
***District (ref–Peri urban)***						
Peripheral	0.005	-0.06–0.07	0.89	0.036	-0.07–0.14	0.524
Central urban	0.13	0.02–0.24	**0.016**	-0.14	-0.32–0.02	0.094
***Gender (ref–Male)***						
Female	0.05	-0.01–0.12	0.13	0.09	-0.01–0.2	0.091
***Main occupation (ref–Office worker)***						
Farmer	-0.17	-0.28-(-0.06)	**0.003**	0.18	0.004–0.35	**0.044**
Unemployed	-0.38	-0.55-(-0.22)	**<0.001**	0.01	-0.27–0.3	0.939
Retired	-0.16	-0.28-(-0.04)	**0.011**	-0.007	-0.2–0.2	0.942
Others	-0.1	-0.23–0.01	0.09	0.03	-0.16–0.2	0.755
***Livestock keeping (ref—Yes)***						
No keeping livestock	-0.04	-0.1–0.04	0.316	-0.03	-0.15–0.08	0.533
***Knowledge score***	-	-	-	0.06	0.05–0.07	**<0.001**

### Sources of information

TV was the most common source of information that participants hear about the MBDs (92.9%), following by loudspeaker (70.6%) and health staffs (54.9%) (Table 5).

**Table 5:** Reported sources of information about MBDs in households with and without livestock in Hanoi, Vietnam.

	*Households with livestock*	*Households without livestock*	All *(n*, *%)*	P value
Never heard	1 (0.4%)	3 (1.1%)	4 (0.8%)	0.63
TV	209 (92.1%)	253 (93.7%)	462 (92.9%)	0.4
Broadcast	89 (39.2%)	115 (42.59%)	204 (41%)	0.44
Loudspeaker	164 (72.2%)	187 (69.2%)	351 (70.6%)	0.46
Internet	61 (26.9%)	88 (32.6%)	149 (29.9%)	0.16
Communication materials	54 (23.8%)	58 (21.5%)	112 (22.5%)	0.54
Health staffs	135 (59.5%)	138 (51.1%)	273 (54.9%)	0.062
Friends	33 (14.5%)	19 (7%)	52 (10.5%)	**0.007**
School	4 (1.7%)	4 (1.5%)	8 (1.6%)	1.00

*“I mainly heard about dengue through TV*, *sometimes I also heard it from the nearby loudspeaker*, *but the sound was not good*. *At that time*, *I had to run out to hear”*

- IDI with household with livestock in Ha Dong district.

School were the least widespread source of information on MBDs with only 1.61% of participants reporting this. There were no differences between households with livestock and without livestock regarding the sources of information they have heard about the MBDs, except from the friends. The percentage of households with livestock heard about the MBDs from their friends was significantly higher than the percentage of households without livestock (p<0.01).

### Perspectives from health staff

According to qualitative interviews with health staff, the MBDs in Hanoi are a big concern, with the focus on dengue transmission. Other diseases including JE and malaria were rare.

*“In Hanoi*, *dengue is the hottest amongst the mosquito-borne diseases*. *Japanese encephalitis cases seldomly appear*. *We found the cases in urban centers last year (2017)* … *About malaria*, *we didn’t find the main vector in recent ten years”*

- IDI with Hanoi Center of Disease Control’s staff

*“Last decade our district recorded many malaria cases*, *but until now*, *it’s almost zero case*. *The main vector for malaria in our district was disappeared; the dengue vectors exist more*. *We have very less of Japanese encephalitis cases”*

- IDI with Chuong My district health center’s staff

From health staff’s perspective, keeping livestock could be a risk factor leading to the increase of the mosquito vector in Hanoi. However, climatic variables or urbanization were considered more important.

*“Livestock attracts the mosquitoes because they serve as blood feeders*. *Mosquito will find them for their meals*. *Also*, *keeping livestock can leave the standing water that is the breeding sites for mosquitoes*. *But climatic factors are more important*. *Besides*, *our district has a fast speed of urbanization with many construction sites*, *many abandoned houses*, *unfinished construction works with many breeding sites for mosquitoes like underground water tanks…”*

- IDI with Ha Dong district health center’s staff

*“It is also a risk factor when people keep livestock nearby their home*, *especially in the past and in the mountainous areas*. *However*, *in my opinion*, *it’s not the main factor but others like temperature*, *humidity*….*”*

- IDI with Hanoi Center of Disease Control’s staff

Also, awareness of the citizens was one of the most vital factors affecting to the morbidity of MBDs. The majority of people in the community were believed to know about MBDs, especially DF. They used a variety of methods to prevent mosquito bites.

*“People have knowledge*, *they know that the dengue mosquito bite people in early morning and in the dusk*. *They use net regularly*, *especially for the children and other methods like electric racket or remove stagnant water preventing the development of larvae*. *I think that they were doing pretty well*. *Many families called the services to spraying the insecticide*. *Or households with livestock used the disinfectant of veterinary sector to spray frequently”*

- IDI with Chuong My district health center’s staff

However, the knowledge and practice of citizen were different depending on their education level, their priority and communication programs in each location. Although, information was provided through different channels, it was not judged to be very efficient.

*“We implemented the communication programs like providing the materials*, *but people didn’t get it because they didn’t read it*, *or they haven’t read it yet*. *They were busy with working or maybe because of their education level”*

- IDI with Ba Dinh district health center’s staff

*“People still have the habit to store water that will be high risk for dengue infection*… *Loudspeaker might be effective at the suburban areas but not effective in the urban centers…Normally*, *it is difficult to approach the people living in the urban centers*, *the health network also couldn’t be covered to reach all households*. *So*, *delivering the communication leaflets faces difficulty that some subjects can’t receive it such as tenants*, *migrants*, *students”*

- IDI with Hanoi Center of Disease Control’s staff

*“The health staffs and local authorities provide a lot of information about dengue fever*, *but they didn’t pay attention about it*. *They still put the unnecessary water containers*, *or leave the garbage around their home”*

- IDI with Ha Dong district health center’s staff

## Discussion

Our findings indicated that the community knowledge about MBDs was at a moderate level with an average score of 18.3 out of 35. This result, however, was slightly better than another study that measured a knowledge score (4.6/19) on DF of 330 patients in a national hospital in Hanoi [16]. However, both studies made the same conclusion about a low basic level of knowledge on the MBDs of Hanoi community [16]. Our result is in agreement with other studies suggesting low knowledge in tropical countries such as Laos [17], Indonesia [18], Malaysia [19], Nepal [20], India [21–23], Yemen [24] and Sri Lanka [25]. Nevertheless, one study conducted on Sint Eustatius island in the Eastern Caribbean and one study conducted in El Salvador showed a higher level of knowledge about MBDs [26, 27]. These differences may reflect a higher knowledge level in some places but could also be due to methodological differences. Both studies in the Caribbean and El Salvador were implemented with only small sample sizes. In addition, the study in the Caribbean collected data shortly after a local chikungunya virus outbreak and the respondents, thus, had likely achieved a better level of knowledge.

The majority of participants (more than 98%) had previously heard about MBDs, especially dengue fever (96%) and malaria (72%). This result was different as compared to studies in India when participants had heard about malaria much more often than other MBDs including dengue, JE, and filariasis [21, 22, 28]. This might due to differences in study time and disease prevalence of each MBD in each region or country. In fact, a later study showed better awareness of dengue as compared to malaria in India [29]. In Hanoi, dengue has been the most common disease transmitted by mosquitoes [13], while malaria, according to qualitative interviews from health staff, has almost disappeared during the recent decades. Zika virus, which has the same vectors as dengue virus, caused a big outbreak in the central and southern regions of Vietnam in 2016 [13]. Therefore, it is understandable that less people (37%) in Hanoi, which is located in the northern part of Vietnam, could mention Zika. JE and filariasis also rarely appear in the study sites, leading to the minor number of participants that have heard about them. Lack of knowledge about JE could have long-term effects on the willingness to participate in the vaccination program against this disease, which was more common before vaccination started [30], and it may be important to make sure the disease is not forgotten. Most respondents could mention breeding sites of mosquitoes. However, a large percentage of the participants (more than 80%) did not know that mosquitoes can breed in clean water, which is actually the preferred site of the dengue vectors. This finding is pretty similar to some studies in India [21, 22] and Bangladesh [31]; but in contrast to others in India and Malaysia that showed more knowledge on this [29, 32]. It should be noticed for the dengue communication program to improve the awareness of citizen on *Aedes* mosquitoes’ breeding sites.

Poor practice against MBDs in both livestock-keeping and non-livestock-keeping communities was found in our study, with the average score of only 3.5 out of 11. This is similar to earlier results in Hanoi that found a low score for preventive practices of 1.5/11 [16], and many other studies from India [21, 22, 29, 33, 34], Indonesia [18], and Yemen [24]; but worse than results of another study in Indonesia which reported a high score of 9.2 out of 11 [35], Malaysia, Laos, and El Salvador [17, 19, 27]. A possible explanation for the poor practice of Hanoi citizens might be the gap in their knowledge and negligent attitude towards MBDs as evident from the quantitative and qualitative findings of this study. This was one of the most important barriers for MBDs prevention and control program in Hanoi that was illustrated in a previous study [5]. Mosquito net is the most important method to prevent mosquito bites. In our study, it was commonly used amongst the participants with more than 90%. This finding is similar to a study from Laos [17], but significantly higher than other studies from the Seremban district, Malaysia [32], India [21, 22, 29, 33, 34] and Yemen [36]. Nevertheless, as mentioned in our qualitative results with the community, people do not always use the mosquito net frequently, posing a risk of getting mosquito bites. The vectors spreading MBDs are, however, very different and using mosquito nets at night is more likely to reduce the risk of contracting malaria and JE, while the risk for dengue infection remains unchanged. Similarly, mostly the dengue vectors will breed in clean water in small containers and are most affected by interventions targeting this. Old knowledge about how to protect themselves against malaria may not be of use when the incidence of malaria has been reduced significantly, but dengue is emerging.

Our study found out that households without livestock had a better knowledge (p<0.05) but poorer practice on MBDs as compared to households with livestock. Households without livestock were assumed to have better living conditions since they had higher average income in our study. Thus, they might have had more chances to approach the sources of health information. Regarding the practice against MBDs, qualitative findings indicated that the households rearing livestock frequently spray insecticide because they received the disinfectants for free of charge from the veterinary staff. Our finding also depicted that they perceived livestock keeping as increasing the risk of mosquito population’s growth higher than non-livestock households (p<0.001). Therefore, they may have better practices regarding prevention and control of MBDs. Qualitative interviews with the health staff and communities indicated that livestock keeping could be a risk factor for MBDs because these activities create more breeding sites for mosquito when feeding water for the livestock or storing water to clean the animal shelter. Pig rearing has been shown to be a risk factor for the vector of JEV [8, 9], while having an animal shelter was associated with a higher incidence of dengue infection [6]. However, another study in Hanoi found no association between livestock keeping and an increasing risk of dengue [11]. From our understanding, this is the first study aiming to measure the knowledge and practice about MBDs in the livestock keeping and non-livestock keeping communities in Vietnam and the world. Further studies should be carried out to explore more about the knowledge and practice on MBDs between these communities and further investigate if livestock keeping could be a risk factor for MBDs such as dengue, malaria, Zika and JE.

The final negative binomial model in our study revealed that people living in central urban districts had better knowledge on MBDs than people living in peri-urban districts. This finding is opposite to a previous smaller study where the participants living in peri-urban district were found to have a better knowledge on dengue than the other district [11]. The explanation for this difference could be due to the sample size and the number of sites in the two studies. Besides, the previous study only focused on the knowledge on dengue fever that was quite familiar among the Hanoi citizens.

As discussed above, the farmers, unemployed and retired people had slightly poorer knowledge on MBDs than the office workers. It is supposed that office workers are those who regularly update their knowledge through the internet while the remaining people have more limitations about time and technology to access health information. Additionally, office workers might have higher level of education which was one of the associated factors for better knowledge on MBDs as shown in our qualitative result of health staff and other studies [16, 18, 24, 32, 35]. One study in Curaҫao also found a positive association of level of education with the increase of accessing information on MBDs [37].

Our study indicated that the farmers had a higher score of using preventive practices on MBDs than office workers. These farmers belonged to the households with livestock that have better practices against MBDs than households without livestock. Both quantitative and qualitative findings indicated that the farmers rearing livestock often used insecticide for their homes and animal shelters. It could be that they are more aware of unhygienic conditions when they keep livestock nearby home, so they have to clean the shelter and house more frequently. In another study in the Binh Thuan province of Vietnam, farmers were at higher risk of getting dengue infection, and therefore they tried preventive methods to protect themselves and their family from MBDs [6]. However, one study showed the opposite result i.e. that farmers were not better in using preventive practice compared as compared to other occupation groups [38]. Our study showed that there was a positive correlation between knowledge and practice on MBDs. The model also indicated that respondents with better knowledge on MBDs had better practices against MBDs. This result was consistent with the result of other studies [16–18, 32]. It is clear that when people perceived a problem adequately, they will tend to execute more correctly.

The most popular source of information of MBDs mentioned by the households in our study was TV. This is similar to other studies in Vietnam [6] and the world [17, 27, 28]. Therefore, this communication channel should be kept updated with a high frequency to the general population. Loudspeakers are also a popular communication channel in Vietnam. It was mentioned by most of participants (70.62%), but its effectiveness was doubted as reported in the interviews with both community and health staff. This issue also was reported in previous study in Vietnam [5] and in Nepal [20]. In our study, the information on MBDs was heard least in school, but it could be that there was a minor percentage of respondents who were students at school at the time of data collection while the older people are not remembering information from school. However, it is noted that students are amongst the high-risk group to get dengue infection in Hanoi [5, 39], and students and pupils have previously been reported to have poor knowledge and practice against MBDs [40], and be less likely to use repellent and bed net to prevent MBDs [38]. Hence, it is recommended that communication campaigns in schools and universities should be organized more regularly. Health staff also frequently mentioned written materials, but these were considered ineffective. There may thus be a need to modernize official information campaigns.

The strength of our study is that we use both quantitative and qualitative components that provided a more comprehensive and exact picture of the knowledge and practice on MBDs, where the qualitative findings elucidated and explained more for the quantitative results. We used the self-designed quantitative questionnaire that were pre-tested and revised from a previous study [11]. The Cronbach Alpha also was used to test the internal consistency of knowledge and practice sections in the questionnaire with high acceptable score.

This study has several limitations. MBDs is a broad term that includes a lot of diseases transmitted from mosquitoes. Therefore, our study could only cover some general characteristics of MBDs’ knowledge and practice, not for each specific MBD. Since our study measured the households’ practice through their response, information bias could happen. In addition, our study focused only on Hanoi, and may not be representative enough to extrapolate to other cities in Vietnam and other countries.

## Conclusion

There was a gap in community knowledge about MBDs, where households with livestock had poorer knowledge than households without livestock. Qualitative findings indicated that livestock-keeping could be related to an increasing risk of mosquito growth and MBDs infections. Findings also revealed a low level of preventive practices against MBDs. Those with better knowledge on MBDs generally also had better practices against MBDs. As compared to office workers, those who were farmers, unemployed and retired people had poorer knowledge on MBDs, but farmers had a higher score of using preventive practices on MBDs than office workers. Television was the most important source of information on MBDs for the households. It is suggested to improve the teaching of MBDs at school and to conduct targeted community campaigns on raising MBDs awareness and changing behavior with the collaboration of health sector and veterinary sector.

## Supporting information

S1 File Questionnaire in English. (DOCX)

S2 File IDI guidelines in English. (DOCX)

S3 File Dataset. (DTA)

S4 File Do file. (DO)
