# Supplementary material for: Knowledge and practice on prevention of mosquito-borne diseases in livestock-keeping and non-livestock-keeping communities in Hanoi city, Vietnam: A mixed-method study
Source: PLoS One. 2021 Feb 4;16(2):e0246032. doi: 10.1371/journal.pone.0246032 (PMC7861445; doi:10.1371/journal.pone.0246032)
Supplement: S1 File — (DOCX) [file pone.0246032.s001.docx]

**Household questionnaire**

**INTRODUCTION**

Good morning / Good afternoon. My name is _________________ and I work for the International Livestock Research Institute (ILRI), which is based in Hanoi. We are conducting a study on how the presence of mosquitoes is affected by the keeping of livestock in urban environments. This study has three parts: 1) a survey to help understand the benefits and risks of urban livestock keeping and the awareness on mosquito-borne diseases; 2) to collect mosquitoes and investigate if the mosquitoes are infected with flaviviruses and, 3) to take blood samples from the livestock at household. We are visiting you because your household has been selected randomly to participate in the study titled “**The association between livestock keeping and mosquito-borne diseases in urban Hanoi”**. If you accept to participate in this study, we will ask you a number of questions related to your household, knowledge and practice of livestock keeping and awareness about mosquito-borne diseases. We will also request you to get the consent to place mosquito traps on your land and take animal’s blood for further laboratory analysis.

**Potential risks**

Your participation in this evaluation has no physical risk. We maintain strict control of all the information gained.

**Potential benefits**

This is an important study to control the burden of neglected tropical diseases such as dengue fever in the community.

**Confidentiality**

The research team promises to respect privacy and confidentiality of your information. This information will only be shared among our research team members, but we will remove all names so that no one will be able to trace back the information to you. If you voluntarily participate in this study, you may withdraw at any time without any consequences of any kind. You may also use the option of removing your data from the study. You may also refuse to answer any questions you don’t want to answer and still remain in the study. The investigator may withdraw you from this research if circumstances arise that warrant doing so. You are not waiving any legal claims, rights or remedies because of your participation in this research study.

If you have any questions now or later you are welcome to call the researchers:

- **Dr Johanna Lindahl**  01675340452 (English)
- **Mr. Nguyen Tien Thang** 0981582712

If you have questions regarding your rights as a research participant, contact:

## **Institutional Review Board**, Room 103 Building A – Hanoi University of Public Health. No 1A, Duc Thang str, Duc Thang ward, Bac Tu Liem district, HaNoi Tel: 024 6266 3024. Email: irb@huph.edu.vn

##

**Questionnaire ID**

ID consists of 5 letters. The first two is district code, 3 remainings are household ID.

District code: **01** – Chuong My, **02** – Dan Phuong, **03** – Bac Tu Liem, **04** – Ha Dong

**05 –** Ba Dinh, **06 –** Cau Giay

| **No** | **A. General information** | | | **Note** |
| --- | --- | --- | --- | --- |
| A1 | Gender | Male  Female | 1  2 |  |
| A2 | Age | _________ |  |  |
| A3 | Highest level of education | College/university  High school  Secondary school  Primary school  No education | 4  3  2  1  0 |  |
| A4 | Marriage status | Single  Married  Separated/Divorced  Widowed  Others: …………...................................... | 1  2  3  4  99 |  |
| A5 | Main occupation | Officer  Farmer  Unemployed  Retired  Others: …………...................................... | 1  2  3  4  99 |  |
| A6 | Number of people in the household | _______________ |  |  |
| A7 | Number of children under 15 in the household | _______________ |  |  |
| A8 | Sources of water using in your family  ***(You can choose more than 1 option)*** | Tap water  Rain water  Rivers/lakes nearby  Well  Others: …………...................................... | 1  2  3  4  5 |  |
| A9 | Type of toilet/sanitation system in your family |  |  |  |
| A10 | Average income per month of your family in total | _______________ VND |  |  |
| **No** | **B. Livestock information** | | | **Note** |
| B0 | Is your family keeping livestock? | Yes  No | 1  0 | **Go to B2** |
| B1 | Why do you not keep livestock?  (for household with no livestock) | Lack of space  No time  Not interested  No/limited knowledge  Concern about hygiene and sanitation  Others: ...................................................... | 1  2  3  4  5  99 | **Go to B10** |
| B2 | Number of livestock last year | Pig ____________________  Goat ____________________  Chicken ____________________  Cattle _____________________  Duck ____________________  Goose ____________________  Others: ____________________ ____________________ |  |  |
| B3 | Number of livestock this year | Pig ____________________  Goat ____________________  Chicken ____________________  Cattle _____________________  Duck ____________________  Goose ____________________  Others: ____________________ ____________________ |  |  |
| B4 | Type of keeping livestock | Indoor  Tied up, partly outdoor  Fenced/shed in outdoor  Free roaming  Others: ...................................................... | 1  2  3  4  99 |  |
| B5 | Who is mainly responsible for keeping livestock in your home | Grandfather/grandmother  Husband  Wife  Children  Others: ...................................................... | 1  2  3  4  99 |  |
| B6 | Have your livestock get any diseases last year? | Yes  No | 1  0 | **Go to B8** |
| B7 | If yes, can you describe the symptoms or name the diseases? | _________________________________ |  |  |
| B8 | How do you usually treat when the livestock got diseases? | _________________________________ |  |  |
| B9 | Why do you keep livestock?  (Benefits)  ***(You can choose more than 1 option)*** | Provide livelihood options  Reduce cost and time of transportation  Food production  Recycling animal waste  Provide the education facilities  Poverty alleviation  Income generation  Pleasures  Others: ...................................................... | 1  2  3  4  5  6  7  8  99 |  |
| B10 | Problems and risks of livestock keeping for all households | Unpleasant smells and noises  Contamination in water resource  Affect the environment/ecosystem  Animal health problems  Feed supply  Theft  Inappropriate waste/manure management  Bring the diseases to human  Low production  Others: ...................................................... | 1  2  3  4  5  6  7  8  9  99 |  |
| B11 | Do you have any pets or other animals in your household? | Cats________  Dogs________  Songbird_______  Other pets___________ |  |  |
| B12 | Do you know any disease that humans can get from animals?  ***(You can choose more than 1 option)*** | Don’t know any  Rabies  Japanese encephalitis  Tuberculosis  Brucellosis  Streptococcus suis  Other_______________ | 0  1  2  3  4  5  99 |  |
| **STT** | **C. Awareness about mosquito-borne diseases** | | | **Note** |
| C1 | Have you heard about diseases being transmitted from mosquitoes to humans | Yes  No | 1  0 | Go to C3 |
| C2 | If yes, can you list the disease(s) that transmitted from mosquitoes  ***(You can choose more than 1 option)*** | Dengue fever  Japanese Encephalitis  Zika  Malaria  Filariasis  Others: ...................................................... | 1  2  3  4  5  99 |  |
| C3 | Can you list breeding sites of mosquitoes?  ***(You can choose more than 1 option)*** | Don’t know  Clean water  Drain/polluted water  Stagnant water containers  Tires  Water tanks  Vase  Bonsai rockery  Others: ...................................................... | 1  2  3  4  5  6  7  8  99 |  |
| C4 | Can you list the risk factors for getting mosquito-borne diseases?  ***(You can choose more than 1 option)*** | Don’t know any  Warm and humid season  High population density  Stagnant water  Livestock keeping  Others: ...................................................... | 0  1  2  3  4  99 |  |
| C5 | Can you list the symptoms while getting mosquito-borne diseases?  ***(You can choose more than 1 option)*** | Don’t know any  High fever  Muscle pains  Nausea/vomiting  Severe headache  Rash  Bleeding  Others: ...................................................... | 0  1  2  3  4  5  6  99 |  |
| C6 | In which season mosquito-borne diseases are highest? | Rainy season  Dry season  Same | 1  2  3 |  |
| C7 | Can you list the ways to prevent yourself from getting mosquito-borne diseases?  ***(You can choose more than 1 option)*** | Don’t know any  Screening of doors/windows  Mosquito repellent creams/ liquid  Mosquito nets  Electric rackets  Mosquito coils / Incense sticks  Long sleeves  Keep lids on water tanks  Chemical in water containers  Anti-mosquito products (e.g. insecticides)  Eliminate breeding sites  Using fish in water containers  Others: ...................................................... | 0  1  2  3  4  5  6  7  8  9  10  11  99 |  |
| C8 | Which personal protection do you use to prevent mosquito-borne diseases?  ***(You can choose more than 1 option)*** | Don’t use any measures  Screening of windows/doors  Mosquito repellent creams/ liquid  Mosquito nets  Electric rackets  Mosquito coils / Incense sticks  Long sleeves  Keep lids on water tanks  Chemical in water containers  Anti-mosquito products (e.g. insecticides)  Eliminate breeding sites  Using fish in water containers  Others: ...................................................... | 0  1  2  3  4  5  6  7  8  9  10  11  99 | **Go to C10** |
| C9 | Frequency of using personal protection to prevent mosquito-borne diseases? | Only at night  Only during the day  Throughout 24h  Few days a week  Others: ...................................................... | 1  2  3  4  99 |  |
| C10 | What are the sources of the information you have heard about the mosquito-borne diseases?  ***(You can choose more than 1 option)*** | Never heard  TV  Broadcast  Loudspeaker  Internet  Communication materials  Health staffs  Friends  School  Other :……………......................... | 0  1  2  3  4  5  6  7  8  99 |  |
| C11 | Which sources of information you like most?  ***(You can choose more than 1 option)*** | TV  Broadcast  Loudspeaker  Internet  Communication materials  Health staffs  Friends  School  Others :……………................................... | 1  2  3  4  5  6  7  8  99 |  |
| C12 | Have you or your family members ever get mosquito-borne diseases during the last 2 years? | Yes  No | 1  0 | **End** |
| C13 | Which disease(s) has/have been diagnosed? | Dengue fever  Japanese Encephalitis  Malaria  No disease was diagnosed  Others :……………................................... | 1  2  3  4  99 |  |
| C14 | Source of treatments for that disease | Public medical system  Private practitioner  Self – medication  No treatment  Others :……………................................... | 1  2  3  0  99 |  |
