# Supplementary material for: Knowledge and practice on prevention of mosquito-borne diseases in livestock-keeping and non-livestock-keeping communities in Hanoi city, Vietnam: A mixed-method study
Source: PLoS One. 2021 Feb 4;16(2):e0246032. doi: 10.1371/journal.pone.0246032 (PMC7861445; doi:10.1371/journal.pone.0246032)
Supplement: S2 File — (DOCX) [file pone.0246032.s002.docx]

# Guideline for in-depth interview with key informants from health sector

1. What is your opinion about the risks and benefits with urban livestock keeping for human health?
2. Which diseases do you think the livestock keeping could contribute to?
3. Can you describe the pattern of vector-borne and mosquito-borne diseases during recent years?
4. Do you think/believe that livestock keeping will increase the risk of presence of mosquito population? Why/why not? If not, which factors does increase the risk of mosquito population?
5. Any documents/policies on prevention and control mosquito-borne diseases/vector-borne diseases are associated with livestock keeping?
6. Can you suggest any methods to reduce the mosquito population in urban areas, especially the household with livestock?

# Guideline for in-depth interview with urban households

1. What are your opinions on the benefits and risks of livestock keeping in urban areas?
2. If you have livestock, why do you have it? What are the difficulties?
3. How do you manage the animal waste?
4. Which diseases do you think the livestock keeping could contribute to?
5. Do you think that livestock keeping in your household, or in close-by households, will increase the risk of presence of mosquito populations at your household? Why/why not? If not, which factors does increase the risk of mosquito populations?
6. Do you think that mosquito-borne diseases are a serious problem in Hanoi? Why/why not?
7. Can you suggest any methods to reduce the mosquito population in urban areas, or around your household?
